# Gastric precancerous diseases classification using CNN with a concise model

**DOI:** 10.1371/journal.pone.0185508

**Published:** 2017-09-26

**Authors:** Xu Zhang, Weiling Hu, Fei Chen, Jiquan Liu, Yuanhang Yang, Liangjing Wang, Huilong Duan, Jianmin Si

**Affiliations:** 1 College of Biomedical Engineering & Instrument Science, Zhejiang University, Hangzhou, China; 2 Key Laboratory of Biomedical Engineering, Ministry of Education, Zhejiang University, Hangzhou, China; 3 Department of Gastroenterology, Sir Run Run Shaw Hospital, Zhejiang University, Hangzhou, China; 4 Institute of Gastroenterology, Zhejiang University, Hangzhou, China; 5 Department of Gastroenterology, Second Affiliated Hospital of Zhejiang University School of Medicine, Hangzhou, China; Harbin Institute of Technology Shenzhen Graduate School, CHINA

## Abstract

Gastric precancerous diseases (GPD) may deteriorate into early gastric cancer if misdiagnosed, so it is important to help doctors recognize GPD accurately and quickly. In this paper, we realize the classification of 3-class GPD, namely, polyp, erosion, and ulcer using convolutional neural networks (CNN) with a concise model called the Gastric Precancerous Disease Network (GPDNet). GPDNet introduces fire modules from SqueezeNet to reduce the model size and parameters about 10 times while improving speed for quick classification. To maintain classification accuracy with fewer parameters, we propose an innovative method called iterative reinforced learning (IRL). After training GPDNet from scratch, we apply IRL to fine-tune the parameters whose values are close to 0, and then we take the modified model as a pretrained model for the next training. The result shows that IRL can improve the accuracy about 9% after 6 iterations. The final classification accuracy of our GPDNet was 88.90%, which is promising for clinical GPD recognition.

## Introduction

Because gastroscopy can observe the gastrointestinal (GI) tract directly, it has been widely applied for GI examinations, which makes it more convenient for doctors to find lesions in the gastric mucosal surface. According to the evolution of cancerization, gastric lesions can be divided into three categories: advanced gastric cancer (AGC), early gastric cancer (EGC), and gastric precancerous disease (GPD). Patients with AGC can rarely be cured, and the 5-year survival rate is no more than 30% [1]. Although the 5-year survival rate of EGC can achieve 90% [2], endoscopic diagnosis of EGC is indeed difficult in most countries [3], and largely depends on the doctors’ experience and the device advancement. Statistics indicate that GPDs, like erosion, polyps, and ulcers, may transform into gastric cancer if they are misdiagnosed or are not treated in time [4]. Based on these facts, it becomes more curial to intervene during the GPD process and develop computer-aided GPD recognition systems to avoid misdiagnosis before GPD transforms into EGC or AGC.

During the past decades, many computational approaches have been proposed for dealing with biomedical data. Liu et al. [5] proposed a novel method that can convert the frequency profile into a series of profile-based proteins and achieved a promising result using support vector machine (SVM). Zheng et al. [6] introduced sparseness with Nonnegative matrix factorization and achieved a better tumor clustering result. A. Tartar et al. [7] have proposed a more robust CAD system with random forest classifier by using morphological features and patient information properties.

Several attempts have also been made in the field of endoscopic image lesion detection using computer vision, especially machine learning. Researchers have realized colonic polyp detection using geometric features, shape features, and texture features [8–10]. Li et al. used a local binary pattern (LBP) and SVM to classify capsule endoscopy images based on wavelet transform [11, 12]. Many works like [13–15] prove the dual-tree complex wavelet transform (DT-CWT) features to be quite effective for distinguishing different types of polyps. X. Shen et al. combined multiscale texture features with color features, which were fed into an AdaBoost classifier to complete gastroscopic image lesion detection [16]. However, traditional machine-learning methods required handcrafted features, which were quite time-consuming and lacked of robustness.

Recently, deep learning using convolutional neural networks (CNNs) has achieved great success in the area of image recognition, including medical image analysis [17–19]. However, models trained from CNNs contain millions of parameters. To avoid overfitting and achieve quick convergence while training, researchers have adopted many effective tricks, including data augmentation [20, 21], ReLU for activation [22], batch normalization, Dropout [23], pretrained model, or transfer learning [24]. Some researchers, who went in a different direction, tried to modify networks for fewer parameters with state-of-the-art performance. SqueezeNet [25] was an outstanding example. SqueezeNet introduced fire modules to take the place of traditional convolutional layers, and it achieved AlexNet-level accuracy on ImageNet with 50 times fewer parameters. Thanks to these contributions, deep learning has also solved some popular issues in medical image analysis [21, 26–28]. However, deep learning shows slow development in endoscopic images. Tajbakhsh, Nima et al. [29, 30] integrate a variety of polyp features into one polyp detection system using an ensemble of CNNs. Ribeiro, Eduardo et al. [31] also realized colonic polyp classification using off-the-shelf CNN architectures. Zhu, Rongsheng et al.[32] combined a CNN for a trainable feature extractor with SVM for a classifier to realize endoscopic image lesion detection. However, these works either focused on colonic images or the networks were off-the-shelf with too many parameters that did not match well with small gastric image datasets.

**Fig 1:**
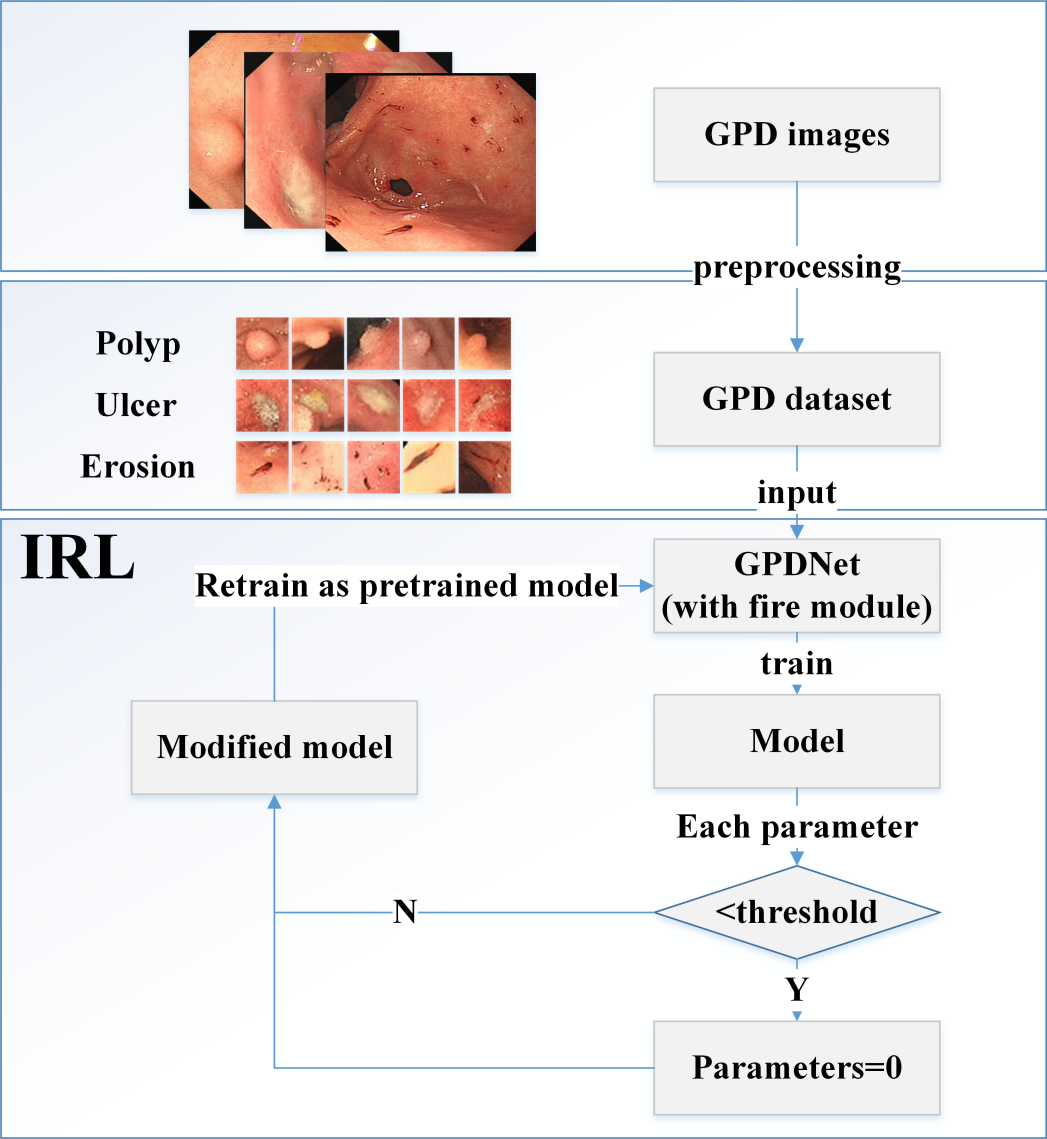
Research flowchart, including IRL algorithm.

To overcome these obstacles, we have proposed a new integrated CNN called the Gastric Precancerous Disease Network (GPDNet); this is a concise network and can recognize GPD precisely. Our GPDNet can help endoscopic doctors decrease misdiagnosis and screen out images containing lesions to alleviate doctors’ workloads. GPDNet introduces fire modules from SqueezeNet to replace traditional convolutional layers and removes the fully connection layers to achieve fully convolutional network (FCN). Based on these efforts, our concise model is smaller and has fewer parameters. To maintain classification accuracy with fewer parameters, we have also proposed a novel fine-tuning algorithm called iterative reinforced learning (IRL). IRL is inspired by the manner in which humans learn and review. The idea of IRL is similar to dense-sparse-dense (DSD) [33]. However, DSD excessively focuses on S (sparse) stage training, which makes the training stage more complex, whereas IRL abandons the S stage, which DSD believes is the most important link. We just reset particular small weights to zero, then we retrain the networks directly. Furthermore, IRL is different from Dropout and DropConnect [34], which randomly drop units (along with their connections) from the neural networks during training. In detail, when we first train the model from scratch, we reset to zero, the parameters in the model that are close to zero. After the modification, we conduct another training. The output is a better model. In the authors’ viewpoint, parameters close to zero are not useful, and need to be reset to zero and relearned. Parameters that are far from zero contribute more to the accuracy of the model. Our research flowchart is shown in Fig 1.

Our contributions to this paper can be summarized as follows: (i) We have pioneered research on the recognition of GPD using convolutional neural networks, proving that deep learning can be applied in gastric images with state-of-the-art results. (ii) We have drawn on the experience of SqueezeNet to reduce the model size and parameters for a concise model, which could save time in gastric image classification and was promising for real-time recognition in gastric videos. (iii) We have proposed IRL to fine-tune the model parameters, which can keep the model’s accuracy with a concise model.

## Materials and methods

In this section, we will first concisely introduce the GPD dataset for training. Then we will establish our GPDNet and present its core components. Finally, we will describe the training stage in detail, including the fine-tuning stage for a more accurate model.

### 2.1. Data acquisition

**Fig 2:**
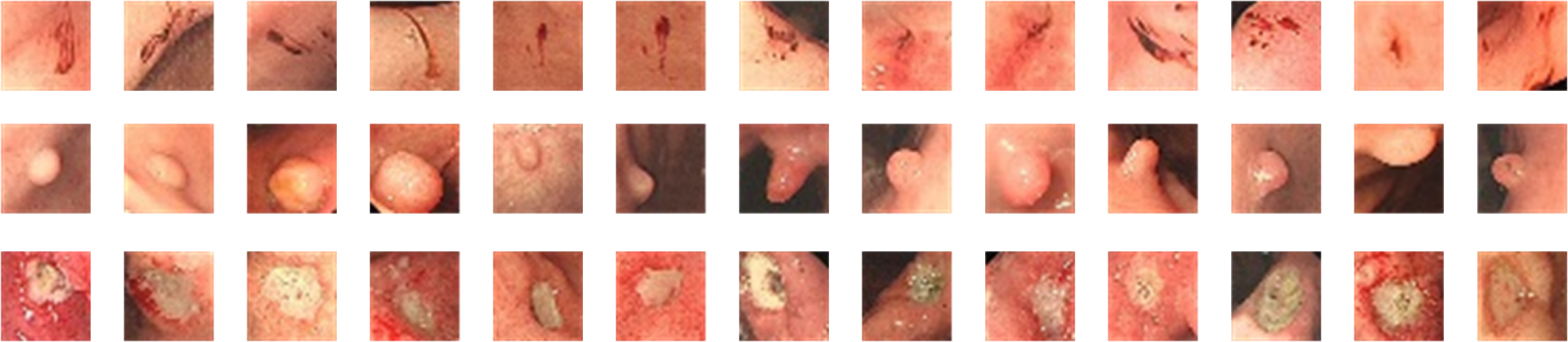
Some samples of GPD training images. The top row denotes erosion lesions. The middle row denotes polyps. The bottom row denotes ulcers. All of them may develop into EGC if they are misdiagnosed during screening.

To evaluate the performance of GPDNet, we collected de-identified gastroscopy images with gastric precancerous diseases from Sir Run Run Shaw Hospital, which all the patients provided written informed consent for their medical images to be published and used in this research. The number of 3-class images was 1331, including 388 images of erosion, 467 images of polyps, and 476 images of ulcer, which were labeled by two professional clinicians with all the gastroscopy images accessed anonymously. The size of the processed images we acquired was 560*475. To expand the image set and reduce the redundant information, we extracted different sized regions of interest (ROI); then we used crop, translation, and other methods to achieve more images. After effective image augmentation, there were 3673 images in total, including 1211 images of erosion, 1218 images of polyps, and 1244 images of ulcer. To simplify our GPDNet for higher speed, we resized all the ROI images to 32*32 (see Fig 1 and Fig 2), so the image size of our GPDNet inputs is 32*32. To avoid overfitting and make the dataset more general, for each class of images, we shuffled them at first, then we selected 300 images randomly as the testing dataset, and the rest images were regarded as the training dataset.

### 2.2. Architecture of GPDNet

**Fig 3:**
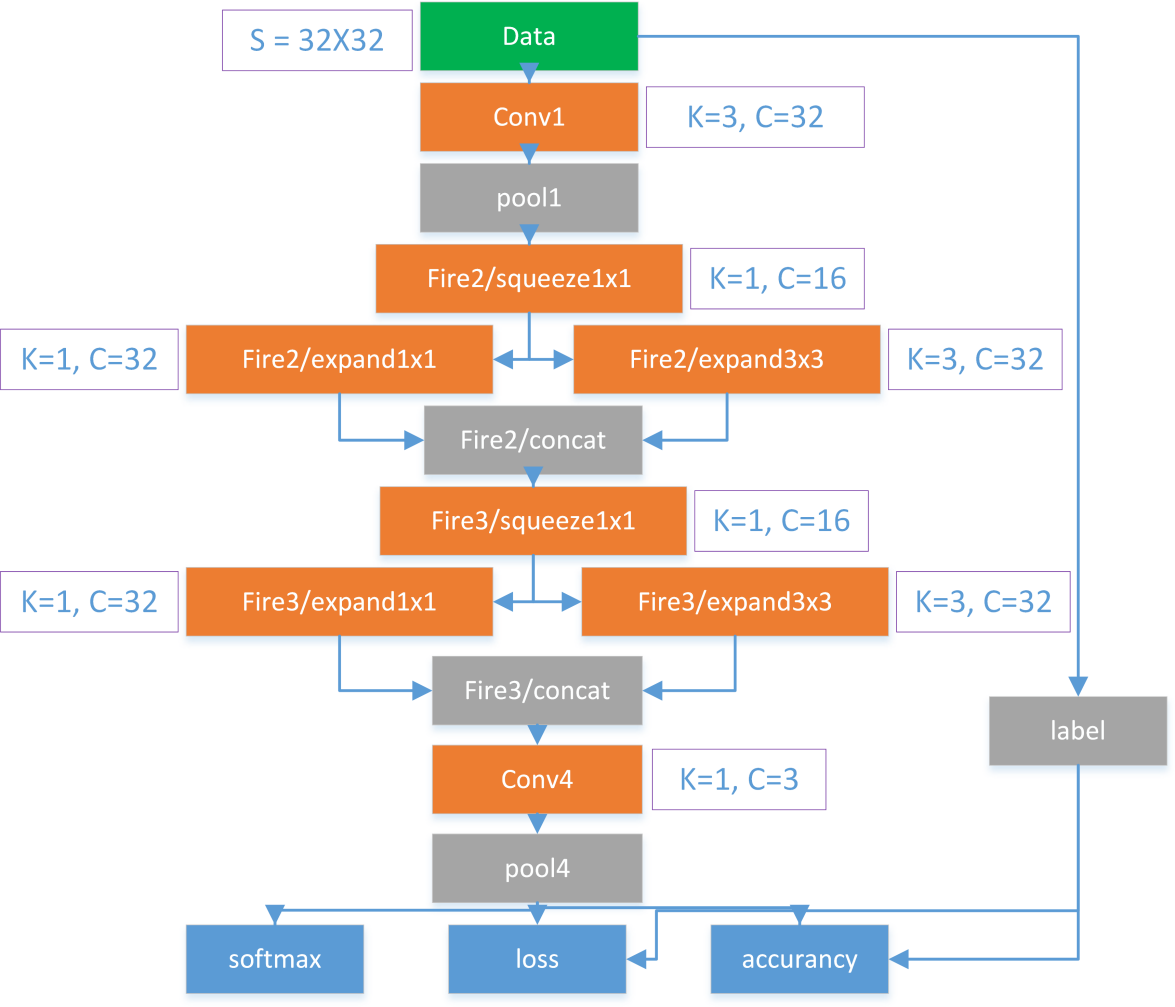
Architecture of GPDNet. K denotes kernel size; C denotes channel or number of feature maps; S denotes input image size.

Fig 3 shows the architecture of GPDNet. Because the input image size of our GPDNet is 32*32, we have applied Alex’s CIFAR-10 network with Caffe style as the basic net. For concise representation, we named this network “cifar-10”. However, cifar-10 is an old-fashioned version. It requires more parameters due to the preserved fully connected layers, which will increase the model size. To decrease the number of parameters and maintain accuracy, we introduced the fire module from SqueezeNet, which is explained clearly in [25], to replace the convolutional layers in cifar-10. A fire module is comprised of a squeezed convolution layer (which has only 1x1 filters), feeding into an expand layer that has a mix of 1x1 and 3x3 convolution filters. Fire modules contribute to the network’s concise models with fewer parameters but higher training speed. In addition, we replaced the fully connected layer in cifar-10 with a convolutional layer for GPDNet, which makes GPDNet a fully convolutional neural network. In Fig 3, the architecture of GPDNet is composed of two traditional convolution layers and two fire modules. In addition, pooling layers are adopted to reduce computation. Because we train GPDNet from scratch, we utilize Xavier to initialize convolutional kernel parameters. To avert the gradient vanishing problem, we chose ReLU as the activate function.

### 2.3. Training and a novel fine-tuning method for GPDNet

As described earlier, we split our dataset *D*{(*X*_*i*_,*l*_*i*_)|*X*_*i*_ ∈ *images*,*l*_*i*_ ∈ *labels*} into a test set containing about 25% of the subjects and the training set containing about 75%. In total, the number of categories L = 3 for classification.

We train the GPDNet using mini-batch stochastic gradient descent. Our loss function can be described as formula (1), where P (l_i_ | X_i_; (W, b)) indicates the probability of X_i_ being correctly classified as l_i_. We aim to solve optimal parameters (W, b) to minimize loss.

$$Loss=\sum_{X_{i},l_{i}\in D}-\log(P(l_{i}|X_{i};(W,b))$$ (1)

The mini-batch size is N = 32. The basic learning rate is 0.001, and we have adopted “step” as our learning rate decay policy. The weight decay is set as 0.9. The training undergoes 50 epochs. After the training is done, the performance of the model acquired is not satisfactory due to using fewer parameters to represent so many images. However, we introduced a novel fine-tuning method IRL to improve the model, which shows that models with fewer parameters can also achieve an outstanding result.

IRL is inspired by the manner in which humans iteratively learn things. In the authors’ opinion, the value of a parameter can reflect its contribution to the model’s accuracy. The larger the value (absolute value) is, the more impact the parameter will have on the model’s accuracy. In other words, the smaller the value is, the less impact the parameter will have. Consider an extreme condition: If the value equals zero, then the corresponding parameter will be regarded as insignificant, and should be retrained or discarded. To conclude, for an arbitrary parameter α in the model, if α is less than the given threshold, it will be set as zero.

$$\forall \alpha \in Model\left\{ \begin{array}{l} \alpha =0,if(|\alpha |\leq threshold).\\ \alpha =\alpha ,otherwise. \end{array}\right.$$ (2)

In this paper, we set the threshold = 0.001. If the model is modified, we will treat this model as the pretrained model for the next training. After several training iterations, the output model should be better.

## Results and discussions

In this section, we will discuss our results from three perspectives. First, we will analyze the correspondence between the model’s accuracy and the value of the parameters. Second, we will discuss whether the threshold value and the number of iterations for the IRL have an impact on the model. Finally, we will compare our CNN architecture with fire modules and without fire modules and analyze GPDNet’s testing results. All the experiments and training tasks were conducted on a Nvidia Tesla K20m GPU (5GB).

### 3.1 Model accuracy versus the value of parameters

**Fig 4:**
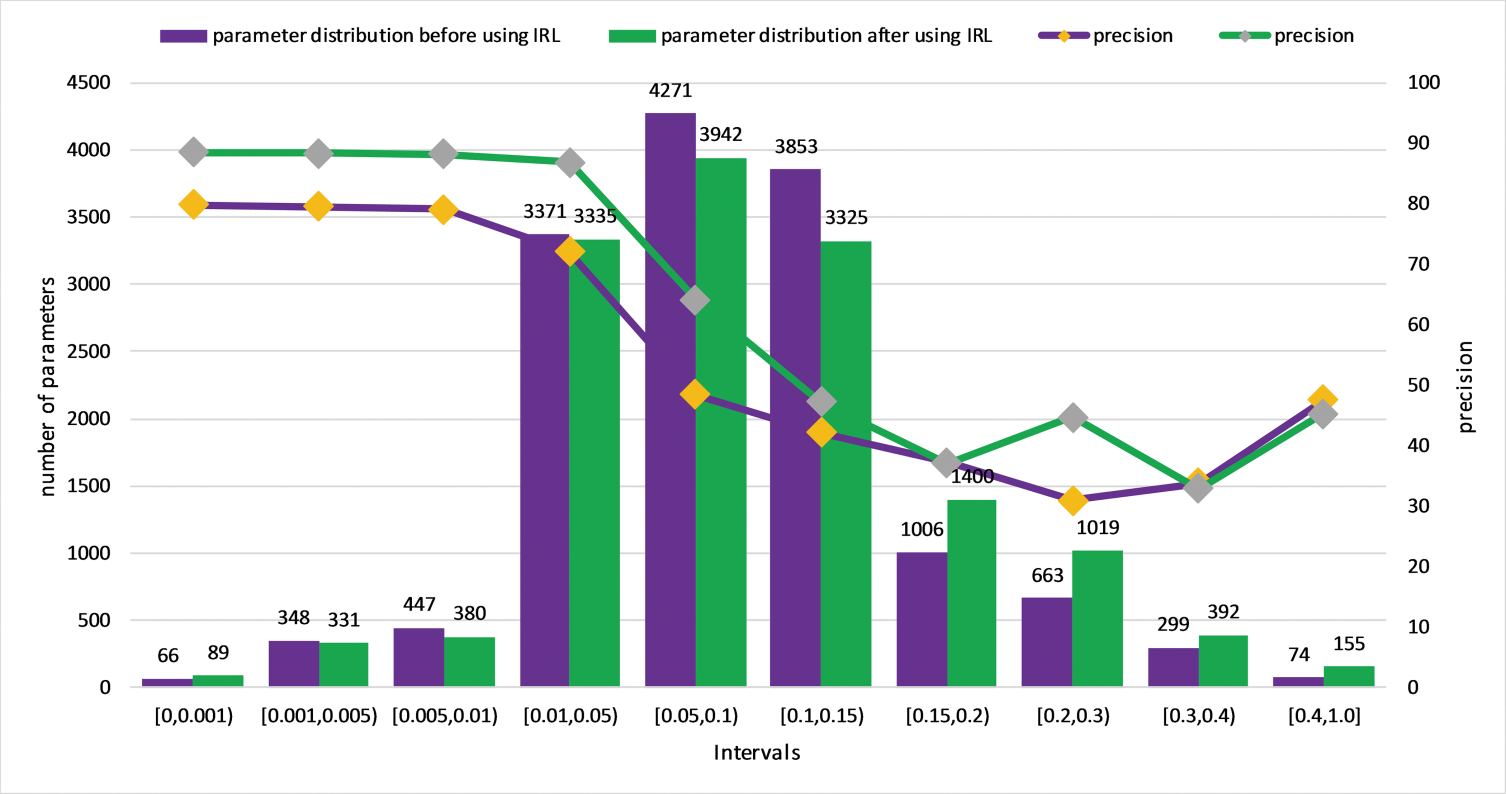
The number of parameters of each range, corresponding to the modified model’s accuracy if we set all the parameters in that range as zero. The purple bar denotes the parameter distribution before using IRL, which corresponds to the purple line. The green bar denotes the parameter distribution after using IRL, which corresponds to the green line. The super-parameters of IRL are threshold = 0.001 and iterations = 5.

Because the value of the model parameters is between -1 and 1, we divided the range [0,1] into ten subranges, [0,0.001), [0.001,0.005), [0.005,0.01), [0.01,0.05), [0.05,0.1), [0.1,0.15), [0.15,0.2), [0.2,0.3), [0.3,0.4), and [0.4,1]. For each subrange, we set all the model parameters of that range as zero, then we tested the modified model’s accuracy with the same test dataset. It’s worth noting that these ten subranges were not divided equally due to the number of parameters distributed unevenly. The result is shown in Fig 4.

From Fig 4, when we have completed IRL, we can find that in [0.01,0.05), there are 3335 parameters. The accuracy drops very little if we set them to zero. However, if we set [0.3,0.4) with 392 parameters or [0.4,1] with 155 parameters to zero, the accuracy drops rapidly. This shows that the value of a parameter has an incredible impact on the model’s accuracy. Specifically, the larger the parameter is, the more impact it has on the model’s accuracy. We can also see an interesting change in the distribution of parameters after using IRL. Even though the number of parameters whose value is less than 0.05 changes slightly, the number of parameters between 0.05 and 0.15 have decreased, which leads to a rapid increase in parameters greater than 0.15. This also shows that the increasing number of large parameters contributes to the improvement of model accuracy.

### 3.2 The choice of iterations and threshold of IRL

To find the optimal threshold and iterations, we set the thresholds as 0.001, 0.005, 0.01, 0.015, 0.05, 0.1, 0.15, 0.2, and 0.3, respectively. For each threshold, we iterated 7 times. Accuracy figures are listed in Table 1. From Table 1, we can determine the following: (1) If we set the threshold too large, the final model accuracy will decrease quickly (even not converge) as expected. The reason is that too large a threshold will break the model’s basic structure. IRL will not be effective anymore in this situation. We should choose a suitable threshold, which can avoid impacting the model’s basic structure. (2) When we have chosen a proper threshold, the model accuracy will tend to relative stability after several iterations, and this “proper threshold” will have little influence on the model accuracy. (3) For GPDNet, when the threshold < 0.05, the model’s basic structure can be preserved, so we choose the IRL threshold = 0.001 in Fig 1 and Table 2.

**Table 1:** Influence on model accuracy of the IRL threshold and iteration times.

Threshold Iteration	0.001	0.005	0.01	0.015	0.05	0.1	0.15	0.2	0.3
0	79.74	79.74	79.74	79.74	79.74	79.74	79.74	79.74	79.74
1	86.1	86.31	86.21	85.88	86.42	86.75	83.73	78.77	**35.67**
2	86.85	87.07	88.64	86.85	87.39	87.50	84.38	48.06	32.97
3	88.36	87.61	87.71	87.61	88.04	87.82	84.81	80.06	32.97
4	88.04	87.61	88.15	88.14	87.93	**88.04**	84.81	82.11	32.97
5	88.15	87.93	**88.58**	88.04	**88.36**	87.61	84.91	46.77	32.97
6	**88.47**	87.93	88.36	**88.90**	88.04	87.93	85.56	82.22	32.97
7	88.47	**88.58**	88.36	88.58	88.25	87.82	**86.42**	**83.19**	32.97

**Table 2:** Comparison between two architectures.

	GPDNet without fire modules	GPDNet with fire modules
Number of fire modules	0	2
Have fully connected layers	Y	N
Number of parameters	144928	**14368**
Model size (caffe model)	568KB	**60KB**
Model’s accuracy without IRL	**87.39%**	79.74%
Model’s accuracy with IRL (iteration = 7, threshold = 0.001)	88.47%	**88.47%**
Model’s best accuracy (%)	**89.22%**	88.90%
Time consuming (classify 900 images)	3.15s	**2.65s**

### 3.3 Comparison between GPDNet with fire modules and without fire modules

Fig 2 displays some samples of GPD training images. To further verify the model trained from GPDNet, we have determined more indicators on the training models between GPDNet with fire modules and without fire modules. The result is shown in Table 2.

Table 2 depicts two GPDNets. The first column denotes GPDNet without fire modules. The second column denotes GPDNet with fire modules. The architecture of GPDNet without fire modules is based on cifar-10. We only change the output number from 10 to 3.

Compared with GPDNet without fire modules, the parameters of GPDNet with fire modules are reduced 10.0 times and the model size is reduced 9.5 times. If we do not use the IRL method, the accuracy is only 79.74%. However, if we apply IRL, the best accuracy can reach 88.9%, which improves about 9%, and the result is comparable to GPDNet without fire modules. Small networks with few parameters will improve computational efficiency, which in turn improves the speed of GPD classification. To confirm this, we classified 900 images, which comprised the test dataset, using the GPDNet models with and without fire modules. The result shows that GPDNet without fire modules costs 3.15 s, while GPDNet with fire modules only requires 2.65 s.

## Conclusion

In this paper, we established a CNN called GPDNet to classify three categories of gastric precancerous diseases. We utilize the fire module as the basic component of the architecture and the size of the concise model is reduced 9.5 times. The number of parameters is reduced 10.0 times. Time consumed in recognition has also decreased due to fewer parameters and smaller model size. However, if we train the model from scratch, the accuracy can only reach 80%, which is not enough for clinical applications. In this paper, we propose IRL to reinforce learning the parameters iteratively. IRL’s threshold should be less than 0.05 and there should be more than four iterations. The result shows that IRL can improve the accuracy to 88.9%, which is comparable with GPDNet without fire modules. Our work is promising for recognition of gastric precancerous disease, which will help doctors avoid misdiagnosis.
